# Supplementary material for: Gene loss and cis-regulatory novelty shaped core histone gene evolution in the apiculate yeast Hanseniaspora uvarum
Source: Genetics. 2024 Jan 25;226(3):iyae008. doi: 10.1093/genetics/iyae008 (PMC10917516; doi:10.1093/genetics/iyae008)
Supplement: iyae008_Supplementary_Data [file iyae008_supplementary_data.zip › Supplemental_Figure_Legends_GENETICS-2023-306264.docx]

**Supplementary Figure Legends**

**Figure S1.** Paralogous gene loss of core histones in *Hanseniaspora*

**A**. Phylogeny of the *Hanseniaspora* and four outgroup species from Steenwyk *et al.* 2019 (*S*. *cerevisiae*, *K*. *marxianus*, *W*. *anomalus*, and *C*. *jadinii*), with the presence and absence of the core histones genes. Purple, the slow-evolving lineage; Green, the fast-evolving lineage.

**B–C**. Synteny analysis of the *HTA2B2* and *HTA1B1* gene clusters. Data for the pre-WGD ancestor and S. cerevisiae were taken from the yeast gene order browser (Byrne and Wolfe 2005), and gene order was manually inferred for *H*. *vineae* (SEL) and *H*. *uvarum* (FEL). Similar results were obtained for the *HHT1F1* and *HHT2F2* gene clusters, in which *HHT1F1* was lost in the FEL (data not shown).

**Figure S2.** Histone divergence in *Hanseniaspora*

**A**. Amino acid alignment of histone H2A from Saccharomycotina species. Sequences were aligned with MAFFT v7 using the “L-INS-I” iterative refinement method (Katoh *et al.* 2019). Alignment was then visualized with the ESPript v3.0 web server using the “Flashy” color scheme, with similarity coloring using “%Equivalent” option and a global score threshold of “0.85” (Robert and Gouet 2014). Secondary structure of H2A was taken from the yeast nucleosome core particle (PDB: 1ID3). For simplicity we only show the alignment for H2A, but the same was done for histones H2B, H3, and H4.

**B.** Maximum likelihood phylogenetic tree of H2A’s from panel A. For simplicity we only show the inferred tree for H2A, but the same was done for histones H2B and a concatenated H3–H4 alignment. ML phylogenetic inferences were carried out using RAxML v8 with the PROTCATGTR model of substitution (Stamatakis 2014). Highlighted in black is the stem branch of *Hanseniaspora*; in green the stem branch of the FEL; and in purple the stem branch of the SEL.

**C**. Branch length comparisons between the three branch as indicated in panel B and all other branches.

**D**. Distribution of branch lengths across all three histone ML phylogenetic trees. In gray all branch lengths. The average branch length (across the three histone ML phylogenetic trees) is then plotted as dotted lines as indicated.

**Figure S3.** Putative Rap1 binding sites in *Hanseniaspora* FEL

**A**. Rap1 binding site (top) aligned to the discovered Rap1-like motif in *Hanseniaspora* FEL species histone control regions.

**B**. Putative Rap1 binding sites are at a defined distance to the Mcm1 biding sites in the *Hanseniaspora* FEL histone control regions.

**C**. Putative Rap1 binding sites are not uniformly distributed across species and across histone control regions, as the majority are found only in H2A-H2B control regions.

**Figure S4.** Mcm1 targets genes are conserved in *H*. *uvarum*

**A**. Dot assays of *H*. *uvarum* histones and histone control regions (HCR) in wildtype and ∆*arg80* strains.

**B**. Forward search for Mcm1 binding sites in orthologs to *S*. *cerevisiae* Mcm1 regulated genes (M-to-G1) in two *Hanseniaspora* species.

**Figure S5.** Editing the Mcm1 MADS-box domain

**A**. Primary sequence and secondary Mcm1 DNA binding domain structure from *H*. *uvarum* (*Huva*) and from *S*. *cerevisiae*’s Arg80 and Mcm1. Modified from Messenguy and Dubois (2003).

**B**. Example transformation from the CRISPR-Cas9 editing. Left, the small guide RNA was transformed without a repair template; Right, sgRNA was transformed with a repair template, resulting in colony growth (sgRNA-resistant clones).

**C**. Genotyping of clones by PCR/digestions. PCR amplicons from the edited clones were digested with diagnostic enzymes EcoNI and KpnI. Successfully edited clones digest with only KpnI, whereas recombinants digest by both, and wildtype digests with EcoNI. Clones were confirmed by Sanger sequencing.

**D**. RNA extractions from wildtype (ScerMcm1) and mutant (HuvaMcm1) strains.

**E**. Pearson correlation coefficients between estimated log transformed transcript abundance from all RNAseq replicates.

**F**. Volcano plot comparing log2 fold change in gene expression between yeast with native *Scer*Mcm1 and strains with *Huva*Mcm1. Significantly dysregulated genes (log2FC>2 or <-2 and p-value < 0.01) are colored depending on the direction of change; upregulated in *Huva*Mcm1, green; and downregulated in *Huva*Mcm1, orange. The *ARG* genes and α-specific genes which are regulated by Arg80/Mcm1 are highlighted.

**G**. Transcriptional changes to genes involved in arginine metabolism (*ARG1*, *ARG3*, *ARG5,6*, and *ARG8*). Log2FC is shown as a green-colored box for each gene, with intensity increasing with upregulation. Additionally, the z-score expression value of each gene is given for the two conditions; left, *Scer*Mcm1; right, *Huva*Mcm1.

**Figure S6.** Cell cycle length in *H*. *uvarum*

**A**. Doubling time inferred from time-lapse movies of *H. uvarum* grown with and without (red x) exposure to the excitation laser.

**B**. Time to cell cycle completion after release from HU arrest, tracking the mother cell and nascent daughter cell. A single mother cell and the subsequent two divisions are shown in the timestamped image sequence. The daughter cell completes its cell cycle ~25 minutes prior to its mother. Scale bar is 5 µm. Time HH:MM:SS.

**C.** Quantification of time to division for 14 mother cells and the subsequent divisions. Statistical significance in the mean difference of time to division was determined with paired t tests between each pair of mother-daughter.

**Video S1.** *H*. *uvarum* H2A-mNG tagged strain growth.

Cells from a mid-log phase culture (A_600_ ≈ 0.6) were secured to the chamber surface and imaged every 2.5 minutes at 30˚C. Only phase contrast images were taken. Scale bar 5 µM; time HH:MM:SS.

**Video S2.** *H*. *uvarum* H2A-mNG tagged strain growth with GFP excitation.

Cells from a mid-log phase culture (A_600_ ≈ 0.6) were secured to the chamber surface and imaged every 5 minutes at 30˚C. Images were acquired from both the RFP channel (mNeonGreen is a LanYFP-derived fluorophore, as such, it is excited by the RFP laser (531 nm), which we found to be less phototoxic than the GFP laser (470 nm)) and phase contrast. Scale bar 10 µM; time HH:MM:SS.

**Video S3.** *H*. *uvarum* H2A-mNG tagged strain growth with GFP excitation after HU release.

Cells were first arrested in HU for 45 minutes and washed 2x in fresh SC medium prior to being secured to the chamber surface. We mixed an equal proportion of untagged and H2A-mNG-tagged cells prior to imaging. Time-lapse images were acquired every 5 minutes at 30˚C. Scale bar 25 µM; time HH:MM:SS.

**Supplemental References**

Byrne K. P., and K. H. Wolfe, 2005 The Yeast Gene Order Browser: combining curated homology and syntenic context reveals gene fate in polyploid species. Genome Res 15: 1456–1461. https://doi.org/10.1101/gr.3672305

Katoh K., J. Rozewicki, and K. D. Yamada, 2019 MAFFT online service: multiple sequence alignment, interactive sequence choice and visualization. Briefings in Bioinformatics 20: 1160–1166. https://doi.org/10.1093/bib/bbx108

Messenguy F., and E. Dubois, 2003 Role of MADS box proteins and their cofactors in combinatorial control of gene expression and cell development. Gene 316: 1–21. https://doi.org/10.1016/S0378-1119(03)00747-9

Robert X., and P. Gouet, 2014 Deciphering key features in protein structures with the new ENDscript server. Nucleic Acids Research 42: W320–W324. https://doi.org/10.1093/nar/gku316

Stamatakis A., 2014 RAxML version 8: a tool for phylogenetic analysis and post-analysis of large phylogenies. Bioinformatics 30: 1312–1313. https://doi.org/10.1093/bioinformatics/btu033
