# Supplementary figures and images for: Gene loss and cis-regulatory novelty shaped core histone gene evolution in the apiculate yeast Hanseniaspora uvarum
Source: Genetics. 2024 Jan 25;226(3):iyae008. doi: 10.1093/genetics/iyae008 (PMC10917516; doi:10.1093/genetics/iyae008)

**A**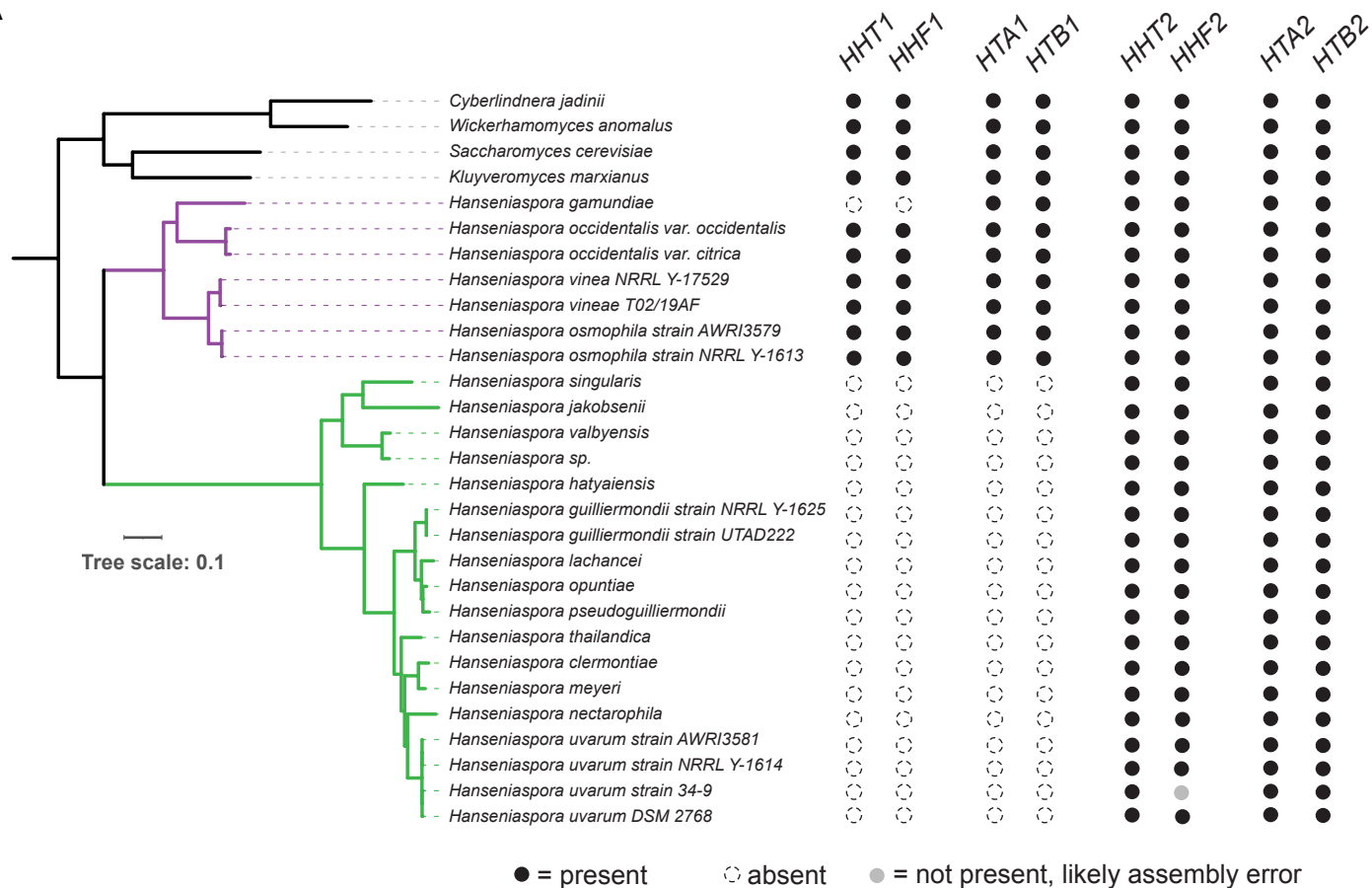**B**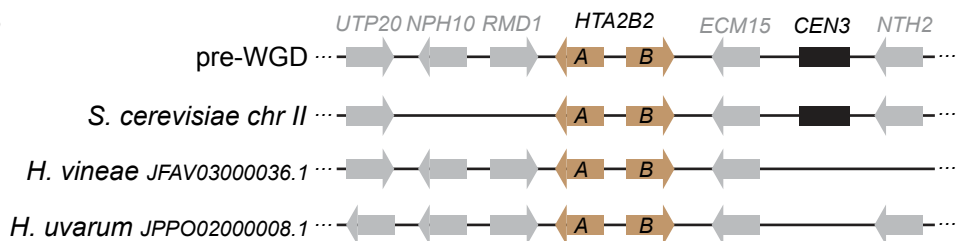**C**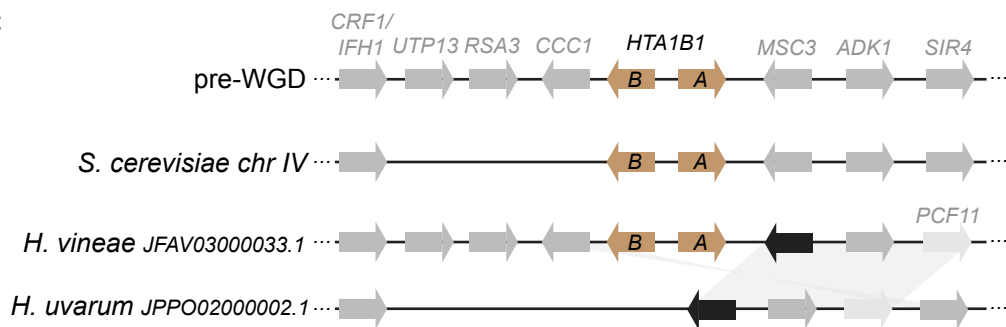

Supplement: iyae008_Supplementary_Data [file iyae008_supplementary_data.zip › Figure_S1_GENETICS-2023-306264.pdf]

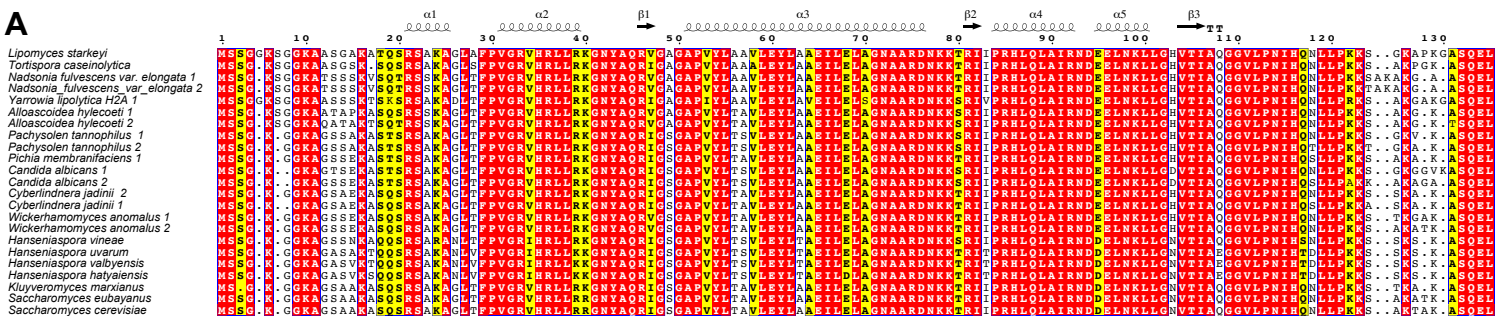

**B** Histone H2A

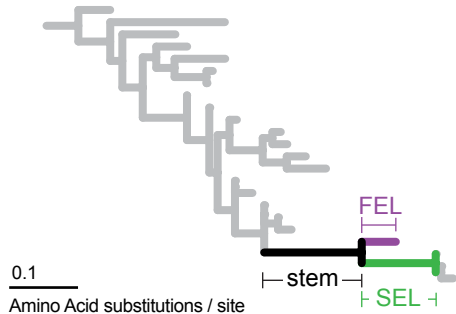

**C**

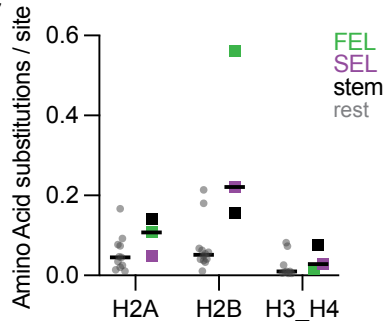

**D**

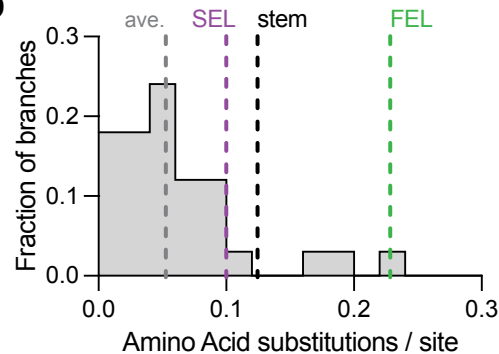

Supplement: iyae008_Supplementary_Data [file iyae008_supplementary_data.zip › Figure_S2_GENETICS-2023-306264.pdf]

**A**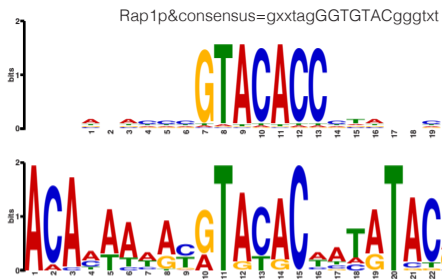**B**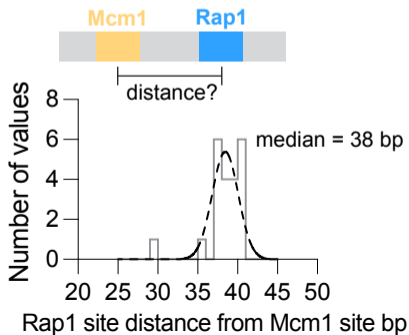**C**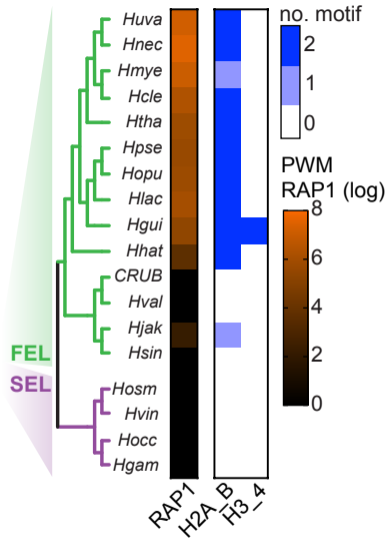

Supplement: iyae008_Supplementary_Data [file iyae008_supplementary_data.zip › Figure_S3_GENETICS-2023-306264.pdf]

**A**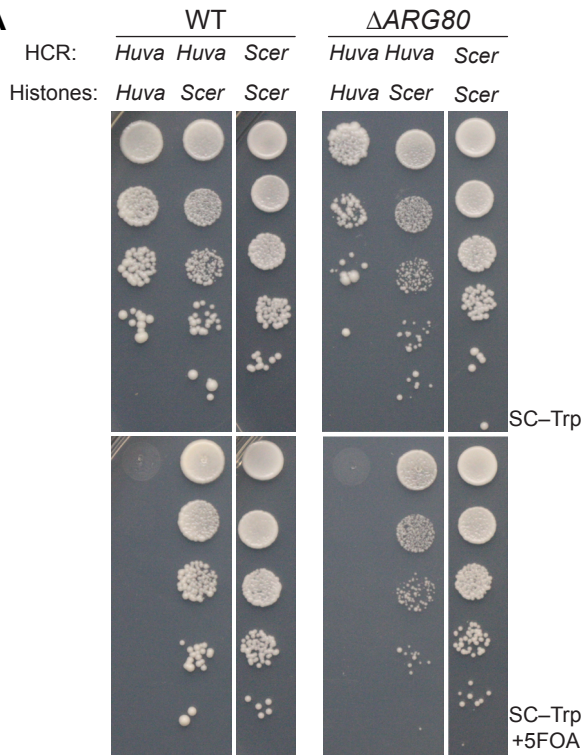**B**

Mcm1 regulated genes **M to G1** in *S. cerevisiae* motif discovery

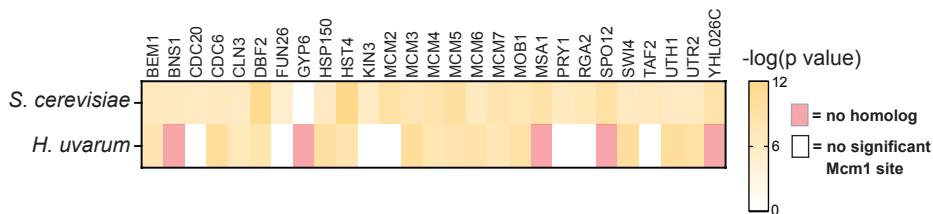

Supplement: iyae008_Supplementary_Data [file iyae008_supplementary_data.zip › Figure_S4_GENETICS-2023-306264.pdf]

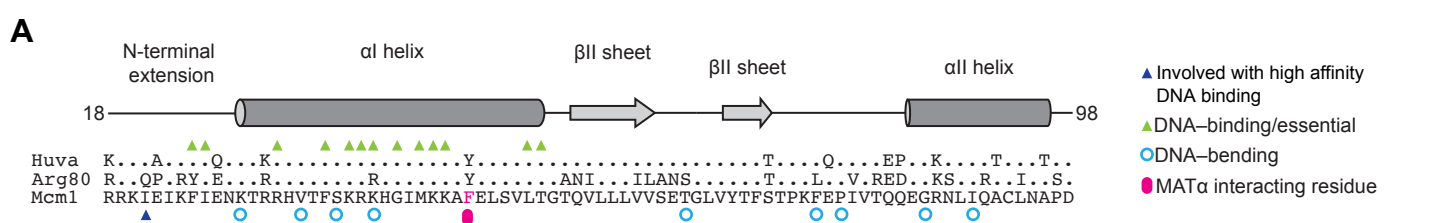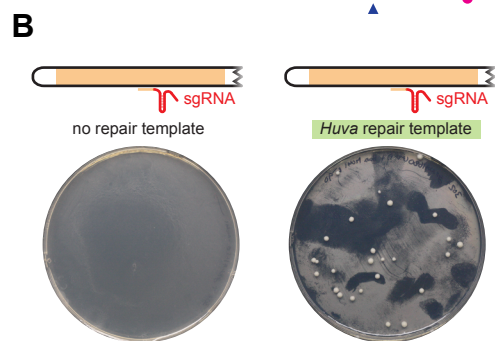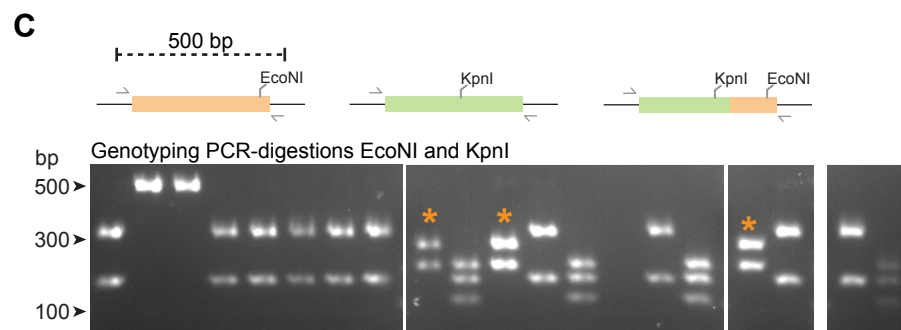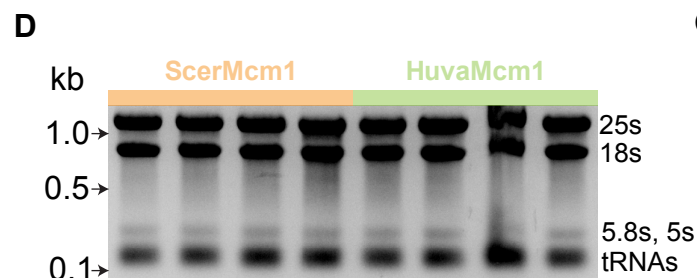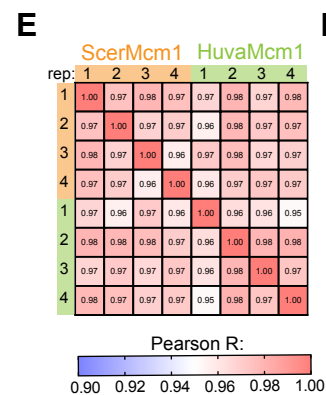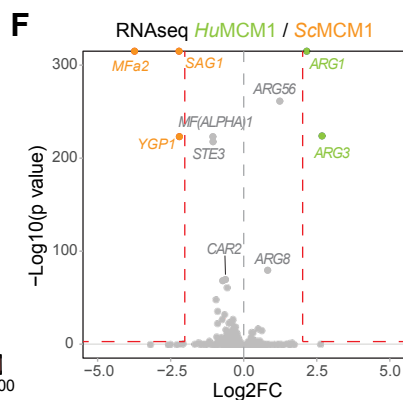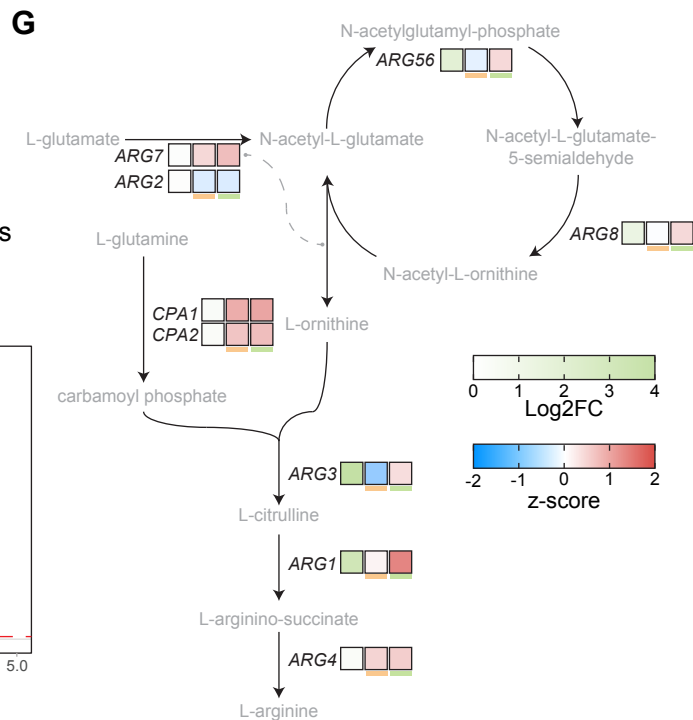

Supplement: iyae008_Supplementary_Data [file iyae008_supplementary_data.zip › Figure_S5_GENETICS-2023-306264.pdf]

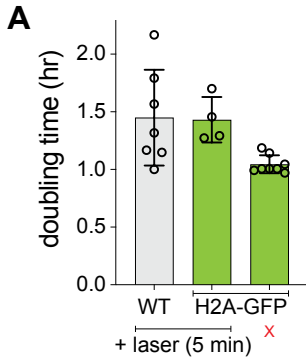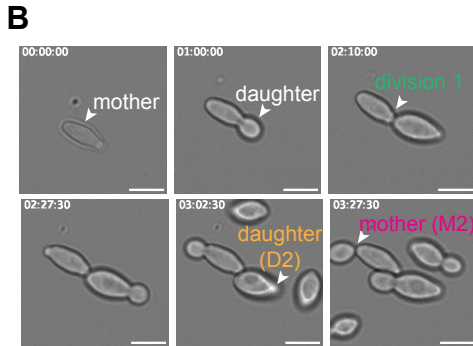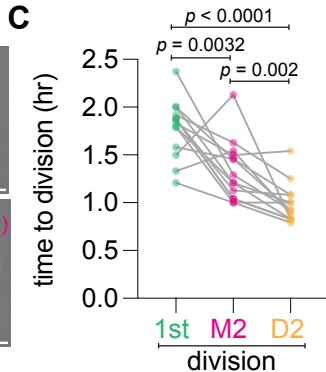

Supplement: iyae008_Supplementary_Data [file iyae008_supplementary_data.zip › Figure_S6_GENETICS-2023-306264.pdf]
